# Supplementary figures and images for: The Shu complex prevents mutagenesis and cytotoxicity of single-strand specific alkylation lesions
Source: eLife. 2021 Nov 1;10:e68080. doi: 10.7554/eLife.68080 (PMC8610418; doi:10.7554/eLife.68080)

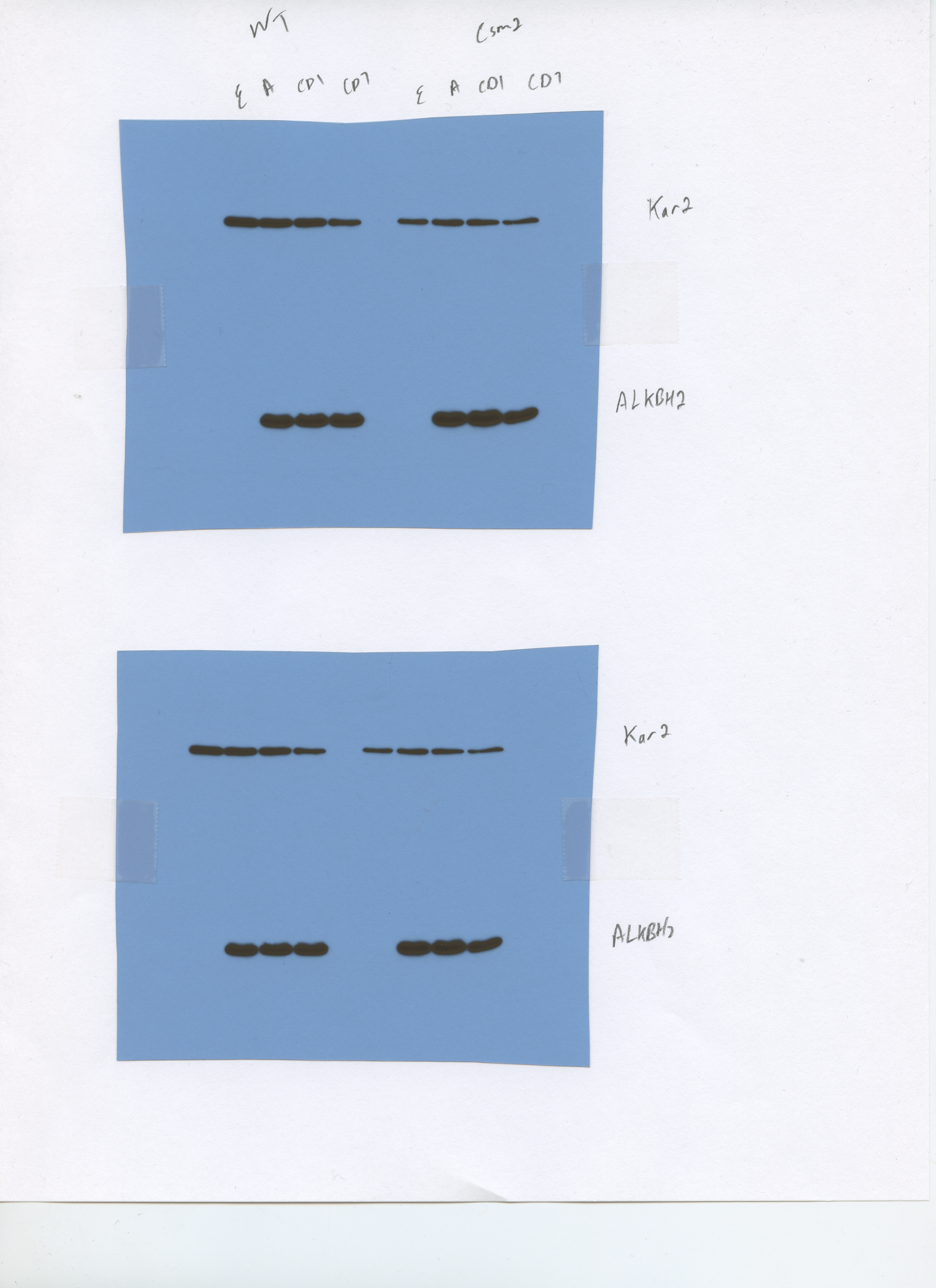

Supplement: Figure 3—figure supplement 1—source data 1. [file elife-68080-fig3-figsupp1-data1.zip › Figure 3-fugure supp;ment 1-source data 1a.tif]

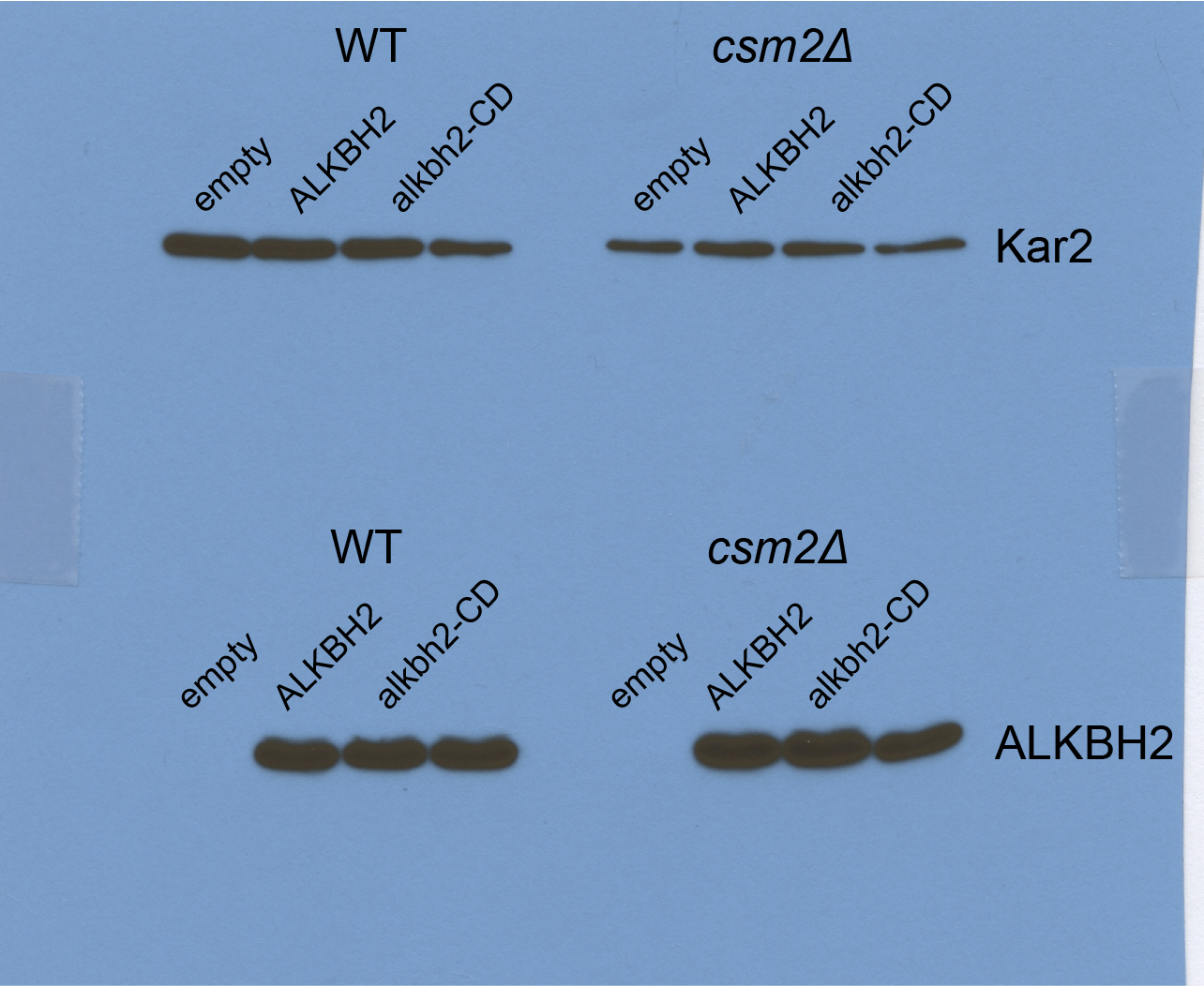

Supplement: Figure 3—figure supplement 1—source data 1. [file elife-68080-fig3-figsupp1-data1.zip › Figure 3-fugure supp;ment 1-source data 1b.png]

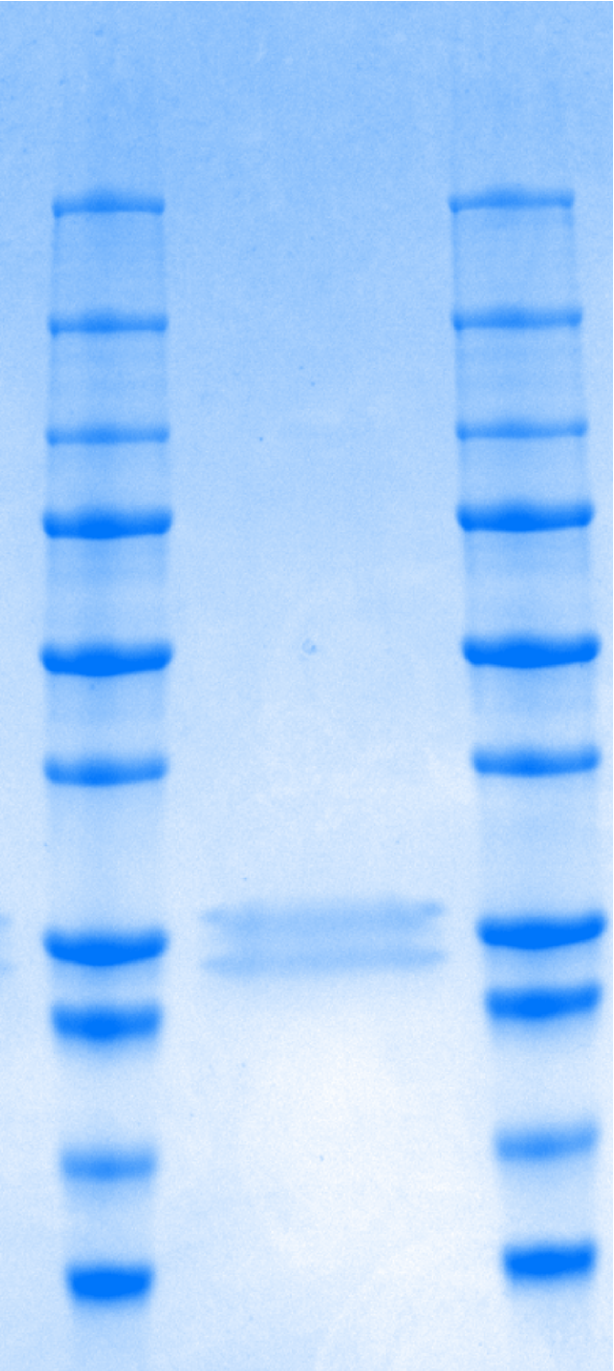

Supplement: Figure 4—source data 2. [file elife-68080-fig4-data2.zip › Figure 4 Source data2a.png]
