# Supplementary material for: The Shu complex prevents mutagenesis and cytotoxicity of single-strand specific alkylation lesions
Source: eLife. 2021 Nov 1;10:e68080. doi: 10.7554/eLife.68080 (PMC8610418; doi:10.7554/eLife.68080)
Supplement: Figure 4—source data 3. [file elife-68080-fig4-data3.zip › 9_2_20215nM1MeACsm2Psy3T4.RTF]

Advanced Reads Report

Report Time : Thu 02 Sep 09:07:24 PM 2021
Batch: C:\Documents and Settings\BEN\Desktop\Sarah\9_2_20215nM1MeACsm2Psy3T4.FBAB
Software Version: 1.1(132)
Operator: 


Instrument Parameters

Instrument                        Cary Eclipse                                                        
Instrument Serial Number          FL0908M003                                                          
Data mode                         Fluorescence                                                        
User Result                       execute("AutoPolarizationCollect.ADL")                              
Ex. Slit (nm)                     10                                                                  
Em. Slit (nm)                     10                                                                  
Ave Time (sec)                    2.0000                                                              
Excitation filter                 Auto                                                                
Emission filter                   Auto                                                                
PMT Voltage (V)                   700                                                                 
Multicell holder                  Multicell                                                           
 Multi zero                       ON                                                                  
Device                                                                                                
 Set temperature (°C)             25.00                                                               
 Monitor                          Block                                                               
Replicates                        OFF                                                                 
Sample averaging                  Duplicate                                                           
Comments:

 
G-Factor
 
 Instrument                5
 Data mode                 Fluorescence
 Ex. Slit (nm)             10
 Em. slit (nm)             10
 Ave. time(s)              2.00000

Ex. WL (nm)   Em. WL (nm)   G-Factor    Int(HV) (a.u)   Int(HH) (a.u.)   
_________________________________________________________________________
     495.00        520.00      1.6235         500.825          308.483   
 
Analysis
Collection time                  9/2/2021 9:07:39 PM                                  
 
Anisotropy
 
     Sample Name         Ex. WL (nm)   Em. WL (nm)      r      G-Factor      Int(VV)      Int(VH)    
_____________________________________________________________________________________________________
  Sample 1                    495.00        520.00      0.04      1.6235       46.989       25.730   
  Sample 1                    495.00        520.00      0.04      1.6235       46.980       25.581   
                                                      0.0409      0.0014         3.36   

  Sample 2                    495.00        520.00      0.04      1.6235       47.567       25.970   
  Sample 2                    495.00        520.00      0.04      1.6235       47.922       26.054   
                                                      0.0417      0.0010         2.48   

  Sample 3                    495.00        520.00      0.05      1.6235       47.434       25.302   
  Sample 3                    495.00        520.00      0.04      1.6235       47.239       25.781   
                                                      0.0451      0.0056        12.45   

  Sample 4                    495.00        520.00      0.04      1.6235       47.137       25.609   
  Sample 4                    495.00        520.00      0.05      1.6235       47.045       25.179   
                                                      0.0453      0.0037         8.11   

  Sample 5                    495.00        520.00      0.05      1.6235       47.432       25.313   
  Sample 5                    495.00        520.00      0.05      1.6235       48.093       25.284   
                                                      0.0515      0.0037         7.18   

  Sample 6                    495.00        520.00      0.07      1.6235       48.506       24.576   
  Sample 6                    495.00        520.00      0.07      1.6235       48.095       24.461   
                                                      0.0664      0.0009         1.43   

  Sample 7                    495.00        520.00      0.08      1.6235       49.180       23.805   
  Sample 7                    495.00        520.00      0.08      1.6235       49.245       23.772   
                                                      0.0838      0.0007         0.81   

  Sample 8                    495.00        520.00      0.09      1.6235       48.978       23.418   
  Sample 8                    495.00        520.00      0.09      1.6235       49.825       23.567   
                                                      0.0896      0.0027         3.05   

  Sample 9                    495.00        520.00      0.10      1.6235       49.571       22.936   
  Sample 9                    495.00        520.00      0.10      1.6235       49.531       22.913   
                                                      0.0995      0.0000         0.05   

  Sample 10                   495.00        520.00      0.16      1.6235       51.404       20.119   
  Sample 10                   495.00        520.00      0.16      1.6235       51.852       20.349   
                                                      0.1601      0.0007         0.43   

  Sample 11                   495.00        520.00      0.17      1.6235       51.696       19.461   
  Sample 11                   495.00        520.00      0.18      1.6235       52.033       19.442   
                                                      0.1764      0.0020         1.11   

  Sample 12                   495.00        520.00      0.20      1.6235       52.281       18.569   
  Sample 12                   495.00        520.00      0.19      1.6235       51.887       18.744   
                                                      0.1935      0.0045         2.31   

  Sample 13                   495.00        520.00      0.20      1.6235       52.148       18.139   
  Sample 13                   495.00        520.00      0.20      1.6235       51.456       18.186   
                                                      0.2014      0.0042         2.10   

  Sample 14                   495.00        520.00      0.21      1.6235       51.747       17.864   
  Sample 14                   495.00        520.00      0.21      1.6235       52.190       18.046   
                                                      0.2069      0.0004         0.21   

  Sample 15                   495.00        520.00      0.22      1.6235       52.039       17.588   
  Sample 15                   495.00        520.00      0.22      1.6235       52.029       17.421   
                                                      0.2169      0.0025         1.14   

Read sequence cancelled

Results Flags Legend
R = Repeat reading               @ = Over-range                                       
