# Supplementary material for: The Shu complex prevents mutagenesis and cytotoxicity of single-strand specific alkylation lesions
Source: eLife. 2021 Nov 1;10:e68080. doi: 10.7554/eLife.68080 (PMC8610418; doi:10.7554/eLife.68080)
Supplement: Figure 4—source data 3. [file elife-68080-fig4-data3.zip › 8_20_2021for5nMWTTplusCsm2Psy3T1.RTF]

Advanced Reads Report

Report Time : Fri 20 Aug 12:30:14 PM 2021
Batch: C:\Documents and Settings\BEN\Desktop\Sarah\8_20_2021for5nMWTTplusCsm2Psy3T1.FBAB
Software Version: 1.1(132)
Operator: 


Instrument Parameters

Instrument                        Cary Eclipse                                                        
Instrument Serial Number          FL0908M003                                                          
Data mode                         Fluorescence                                                        
User Result                       execute("AutoPolarizationCollect.adl")                              
Ex. Slit (nm)                     10                                                                  
Em. Slit (nm)                     10                                                                  
Ave Time (sec)                    2.0000                                                              
Excitation filter                 Auto                                                                
Emission filter                   Auto                                                                
PMT Voltage (V)                   High                                                                
Multicell holder                  Multicell                                                           
 Multi zero                       ON                                                                  
Device                                                                                                
 Set temperature (°C)             25.00                                                               
 Monitor                          Block                                                               
Replicates                        OFF                                                                 
Sample averaging                  Duplicate                                                           
Comments:

 
G-Factor
 
 Instrument                5
 Data mode                 Fluorescence
 Ex. Slit (nm)             10
 Em. slit (nm)             10
 Ave. time(s)              2.00000

Ex. WL (nm)   Em. WL (nm)   G-Factor    Int(HV) (a.u)   Int(HH) (a.u.)   
_________________________________________________________________________
     495.00        520.00      1.0000        1000.000         1000.000   
 
Analysis
Collection time                  8/20/2021 12:30:40 PM                                
 
Anisotropy
 
     Sample Name         Ex. WL (nm)   Em. WL (nm)      r      G-Factor      Int(VV)      Int(VH)    
_____________________________________________________________________________________________________
  Sample 1                    495.00        520.00      0.21      1.0000      215.743      121.540   
  Sample 1                    495.00        520.00      0.21      1.0000      217.599      120.021   
                                                      0.2093      0.0056         2.67   

  Sample 2                    495.00        520.00      0.21      1.0000      210.206      116.726   
  Sample 2                    495.00        520.00      0.21      1.0000      210.852      116.255   
                                                      0.2120      0.0019         0.89   

  Sample 3                    495.00        520.00      0.22      1.0000      210.879      114.651   
  Sample 3                    495.00        520.00      0.21      1.0000      208.482      114.723   
                                                      0.2164      0.0032         1.47   

  Sample 4                    495.00        520.00      0.22      1.0000      206.289      112.930   
  Sample 4                    495.00        520.00      0.21      1.0000      207.096      114.178   
                                                      0.2147      0.0019         0.87   

  Sample 5                    495.00        520.00      0.22      1.0000      206.895      113.277   
  Sample 5                    495.00        520.00      0.22      1.0000      206.275      112.555   
                                                      0.2166      0.0009         0.41   

  Sample 6                    495.00        520.00      0.22      1.0000      206.915      111.068   
  Sample 6                    495.00        520.00      0.23      1.0000      208.419      109.648   
                                                      0.2272      0.0053         2.34   

  Sample 7                    495.00        520.00      0.23      1.0000      206.709      109.075   
  Sample 7                    495.00        520.00      0.23      1.0000      205.190      108.491   
                                                      0.2294      0.0005         0.23   

  Sample 8                    495.00        520.00      0.24      1.0000      205.097      105.567   
  Sample 8                    495.00        520.00      0.24      1.0000      205.201      104.716   
                                                      0.2407      0.0023         0.95   

  Sample 9                    495.00        520.00      0.24      1.0000      204.454      104.918   
  Sample 9                    495.00        520.00      0.24      1.0000      203.861      103.464   
                                                      0.2423      0.0029         1.21   

  Sample 10                   495.00        520.00      0.29      1.0000      211.619       96.224   
  Sample 10                   495.00        520.00      0.29      1.0000      210.942       95.417   
                                                      0.2866      0.0014         0.48   

  Sample 11                   495.00        520.00      0.31      1.0000      195.001       82.981   
  Sample 11                   495.00        520.00      0.31      1.0000      197.714       83.665   
                                                      0.3114      0.0015         0.47   

  Sample 12                   495.00        520.00      0.31      1.0000      197.371       83.757   
  Sample 12                   495.00        520.00      0.31      1.0000      195.188       83.419   
                                                      0.3101      0.0019         0.60   

  Sample 13                   495.00        520.00      0.36      1.0000      214.323       79.176   
  Sample 13                   495.00        520.00      0.37      1.0000      216.272       79.078   
                                                      0.3645      0.0027         0.73   

  Sample 14                   495.00        520.00      0.33      1.0000      192.774       77.618   
Read sequence cancelled

Results Flags Legend
R = Repeat reading               @ = Over-range                                       
