# Supplementary material for: The Shu complex prevents mutagenesis and cytotoxicity of single-strand specific alkylation lesions
Source: eLife. 2021 Nov 1;10:e68080. doi: 10.7554/eLife.68080 (PMC8610418; doi:10.7554/eLife.68080)
Supplement: Figure 4—source data 3. [file elife-68080-fig4-data3.zip › 8_11_20215nMWTTCsm2Psy3T1forreal.RTF]

Advanced Reads Report

Report Time : Wed 11 Aug 04:39:42 PM 2021
Batch: C:\Documents and Settings\BEN\Desktop\Sarah\poo43.FBAB
Software Version: 1.1(132)
Operator: 


Instrument Parameters

Instrument                        Cary Eclipse                                                        
Instrument Serial Number          FL0908M003                                                          
Data mode                         Fluorescence                                                        
User Result                       execute("AutoPolarizationCollect.ADL")                              
Ex. Slit (nm)                     10                                                                  
Em. Slit (nm)                     10                                                                  
Ave Time (sec)                    2.0000                                                              
Excitation filter                 Auto                                                                
Emission filter                   Auto                                                                
PMT Voltage (V)                   High                                                                
Multicell holder                  Multicell                                                           
 Multi zero                       ON                                                                  
Device                                                                                                
 Set temperature (°C)             25.00                                                               
 Monitor                          Block                                                               
Replicates                        2                                                                   
Sample averaging                  OFF                                                                 
Comments:

Analysis
Collection time                  8/11/2021 4:39:42 PM                                 
 
Anisotropy
 
     Sample Name         Ex. WL (nm)   Em. WL (nm)      r      G-Factor      Int(VV)      Int(VH)    
_____________________________________________________________________________________________________
  Sample 1                    495.00        520.00      0.10      1.4005       30.470       16.290   
  Sample 1                    495.00        520.00      0.10      1.4005       30.308       16.218   
                                            0.1004    0.0002        0.24   

  Sample 2                    495.00        520.00      0.11      1.4005       29.716       15.628   
  Sample 2                    495.00        520.00      0.11      1.4005       30.027       15.625   
                                            0.1084    0.0027        2.49   

  Sample 3                    495.00        520.00      0.13      1.4005       29.743       14.726   
  Sample 3                    495.00        520.00      0.12      1.4005       29.644       14.864   
                                            0.1262    0.0033        2.59   

  Sample 4                    495.00        520.00      0.13      1.4005       29.488       14.461   
  Sample 4                    495.00        520.00      0.13      1.4005       29.885       14.652   
                                            0.1320    0.0001        0.05   

  Sample 5                    495.00        520.00      0.14      1.4005       29.146       14.016   
  Sample 5                    495.00        520.00      0.14      1.4005       29.046       13.897   
                                            0.1401    0.0013        0.94   

  Sample 6                    495.00        520.00      0.17      1.4005       28.842       12.811   
  Sample 6                    495.00        520.00      0.15      1.4005       28.402       13.229   
                                            0.1596    0.0124        7.77   

  Sample 7                    495.00        520.00      0.15      1.4005       28.736       13.288   
  Sample 7                    495.00        520.00      0.16      1.4005       28.915       12.982   
                                            0.1590    0.0077        4.85   

  Sample 8                    495.00        520.00      0.18      1.4005       27.940       11.988   
  Sample 8                    495.00        520.00      0.17      1.4005       27.954       12.325   
                                            0.1762    0.0072        4.06   

  Sample 9                    495.00        520.00      0.19      1.4005       26.923       11.258   
  Sample 9                    495.00        520.00      0.18      1.4005       27.196       11.793   
                                            0.1841    0.0096        5.19   

  Sample 10                   495.00        520.00      0.20      1.4005       26.515       10.917   
  Sample 10                   495.00        520.00      0.19      1.4005       26.068       10.988   
                                            0.1922    0.0062        3.22   

  Sample 11                   495.00        520.00      0.23      1.4005       26.466       10.034   
  Sample 11                   495.00        520.00      0.23      1.4005       27.140       10.170   
                                            0.2296    0.0031        1.35   

  Sample 12                   495.00        520.00      0.23      1.4005       25.941        9.856   
  Sample 12                   495.00        520.00      0.22      1.4005       26.130        9.976   
                                            0.2257    0.0013        0.57   

  Sample 13                   495.00        520.00      0.24      1.4005       26.383        9.675   
  Sample 13                   495.00        520.00      0.23      1.4005       25.631        9.660   
                                            0.2348    0.0072        3.08   

  Sample 14                   495.00        520.00      0.24      1.4005       26.318        9.747   
  Sample 14                   495.00        520.00      0.24      1.4005       25.539        9.411   
                                            0.2372    0.0013        0.57   

Read sequence cancelled

Results Flags Legend
R = Repeat reading               @ = Over-range                                       
