# Supplementary material for: The Shu complex prevents mutagenesis and cytotoxicity of single-strand specific alkylation lesions
Source: eLife. 2021 Nov 1;10:e68080. doi: 10.7554/eLife.68080 (PMC8610418; doi:10.7554/eLife.68080)
Supplement: Figure 4—source data 3. [file elife-68080-fig4-data3.zip › 9_2_20215nM3MeCCsm2Psy3T1.RTF]

Advanced Reads Report

Report Time : Thu 02 Sep 02:55:08 PM 2021
Batch: C:\Documents and Settings\BEN\Desktop\Sarah\9_2_20215nM3MeCCsm2Psy3T1.FBAB
Software Version: 1.1(132)
Operator: 


Instrument Parameters

Instrument                        Cary Eclipse                                                        
Instrument Serial Number          FL0908M003                                                          
Data mode                         Fluorescence                                                        
User Result                       execute("AutoPolarizationCollect.ADL")                              
Ex. Slit (nm)                     10                                                                  
Em. Slit (nm)                     10                                                                  
Ave Time (sec)                    2.0000                                                              
Excitation filter                 Auto                                                                
Emission filter                   Auto                                                                
PMT Voltage (V)                   700                                                                 
Multicell holder                  Multicell                                                           
 Multi zero                       ON                                                                  
Device                                                                                                
 Set temperature (°C)             25.00                                                               
 Monitor                          Block                                                               
Replicates                        OFF                                                                 
Sample averaging                  Duplicate                                                           
Comments:

 
G-Factor
 
 Instrument                5
 Data mode                 Fluorescence
 Ex. Slit (nm)             10
 Em. slit (nm)             10
 Ave. time(s)              2.00000

Ex. WL (nm)   Em. WL (nm)   G-Factor    Int(HV) (a.u)   Int(HH) (a.u.)   
_________________________________________________________________________
     495.00        520.00      1.6291         517.352          317.572   
 
Analysis
Collection time                  9/2/2021 2:55:28 PM                                  
 
Anisotropy
 
     Sample Name         Ex. WL (nm)   Em. WL (nm)      r      G-Factor      Int(VV)      Int(VH)    
_____________________________________________________________________________________________________
  Sample 1                    495.00        520.00      0.03      1.6291       49.242       27.597   
  Sample 1                    495.00        520.00      0.03      1.6291       49.414       27.373   
                                                      0.0328      0.0028         8.63   

  Sample 2                    495.00        520.00      0.03      1.6291       48.939       27.259   
  Sample 2                    495.00        520.00      0.04      1.6291       49.526       27.228   
                                                      0.0351      0.0032         9.03   

  Sample 3                    495.00        520.00      0.04      1.6291       48.976       27.013   
  Sample 3                    495.00        520.00      0.04      1.6291       49.517       27.023   
                                                      0.0381      0.0026         6.80   

  Sample 4                    495.00        520.00      0.04      1.6291       49.331       26.872   
  Sample 4                    495.00        520.00      0.04      1.6291       49.412       26.742   
                                                      0.0417      0.0016         3.79   

  Sample 5                    495.00        520.00      0.04      1.6291       48.513       26.282   
  Sample 5                    495.00        520.00      0.05      1.6291       48.920       26.149   
                                                      0.0448      0.0033         7.34   

  Sample 6                    495.00        520.00      0.06      1.6291       48.056       24.966   
  Sample 6                    495.00        520.00      0.06      1.6291       48.413       24.851   
                                                      0.0592      0.0030         5.04   

  Sample 7                    495.00        520.00      0.07      1.6291       47.498       23.927   
  Sample 7                    495.00        520.00      0.07      1.6291       47.763       23.983   
                                                      0.0685      0.0008         1.16   

  Sample 8                    495.00        520.00      0.07      1.6291       47.590       23.632   
  Sample 8                    495.00        520.00      0.08      1.6291       47.408       23.220   
                                                      0.0754      0.0035         4.58   

  Sample 9                    495.00        520.00      0.08      1.6291       47.568       23.059   
  Sample 9                    495.00        520.00      0.08      1.6291       47.213       23.106   
                                                      0.0798      0.0024         2.99   

  Sample 10                   495.00        520.00      0.13      1.6291       47.922       20.218   
  Sample 10                   495.00        520.00      0.12      1.6291       47.556       20.499   
                                                      0.1278      0.0055         4.34   

  Sample 11                   495.00        520.00      0.15      1.6291       47.225       18.797   
  Sample 11                   495.00        520.00      0.15      1.6291       47.057       18.942   
                                                      0.1510      0.0029         1.95   

  Sample 12                   495.00        520.00      0.17      1.6291       46.621       17.903   
  Sample 12                   495.00        520.00      0.17      1.6291       46.772       17.665   
                                                      0.1694      0.0044         2.57   

  Sample 13                   495.00        520.00      0.17      1.6291       45.498       17.101   
  Sample 13                   495.00        520.00      0.17      1.6291       44.953       17.198   
                                                      0.1710      0.0046         2.72   

  Sample 14                   495.00        520.00      0.18      1.6291       44.274       16.306   
  Sample 14                   495.00        520.00      0.17      1.6291       44.028       16.589   
                                                      0.1776      0.0060         3.37   

Read sequence cancelled

Results Flags Legend
R = Repeat reading               @ = Over-range                                       
