# Supplementary material for: The Shu complex prevents mutagenesis and cytotoxicity of single-strand specific alkylation lesions
Source: eLife. 2021 Nov 1;10:e68080. doi: 10.7554/eLife.68080 (PMC8610418; doi:10.7554/eLife.68080)
Supplement: Figure 4—source data 3. [file elife-68080-fig4-data3.zip › 8_19_2021for5nMWTTplusCsm2Psy3T2.RTF]

Advanced Reads Report

Report Time : Thu 19 Aug 02:51:42 PM 2021
Batch: C:\Documents and Settings\BEN\Desktop\Sarah\8_19_2021for5nMWTTplusCsm2Psy3T2.FBAB
Software Version: 1.1(132)
Operator: 


Instrument Parameters

Instrument                        Cary Eclipse                                                        
Instrument Serial Number          FL0908M003                                                          
Data mode                         Fluorescence                                                        
User Result                       execute("AutoPolarizationCollect.ADL")                              
Ex. Slit (nm)                     10                                                                  
Em. Slit (nm)                     10                                                                  
Ave Time (sec)                    2.0000                                                              
Excitation filter                 Auto                                                                
Emission filter                   Auto                                                                
PMT Voltage (V)                   700                                                                 
Multicell holder                  Multicell                                                           
 Multi zero                       ON                                                                  
Device                                                                                                
 Set temperature (°C)             25.00                                                               
 Monitor                          Block                                                               
Replicates                        OFF                                                                 
Sample averaging                  Duplicate                                                           
Comments:

 
G-Factor
 
 Instrument                5
 Data mode                 Fluorescence
 Ex. Slit (nm)             10
 Em. slit (nm)             10
 Ave. time(s)              2.00000

Ex. WL (nm)   Em. WL (nm)   G-Factor    Int(HV) (a.u)   Int(HH) (a.u.)   
_________________________________________________________________________
     495.00        520.00      1.6255         570.326          350.861   
 
Analysis
Collection time                  8/19/2021 2:54:31 PM                                 
 
Anisotropy
 
     Sample Name         Ex. WL (nm)   Em. WL (nm)      r      G-Factor      Int(VV)      Int(VH)    
_____________________________________________________________________________________________________
  Sample 1                    495.00        520.00      0.03      1.6255       53.512       29.981   
  Sample 1                    495.00        520.00      0.03      1.6255       53.557       29.845   
                                                      0.0326      0.0013         4.04   

  Sample 2                    495.00        520.00      0.04      1.6255       52.734       28.931   
  Sample 2                    495.00        520.00      0.04      1.6255       52.721       28.858   
                                                      0.0393      0.0006         1.42   

  Sample 3                    495.00        520.00      0.05      1.6255       52.045       27.888   
  Sample 3                    495.00        520.00      0.05      1.6255       51.829       27.785   
                                                      0.0470      0.0001         0.23   

  Sample 4                    495.00        520.00      0.05      1.6255       51.539       27.459   
  Sample 4                    495.00        520.00      0.05      1.6255       52.067       27.429   
                                                      0.0510      0.0028         5.45   

  Sample 5                    495.00        520.00      0.05      1.6255       51.085       26.832   
  Sample 5                    495.00        520.00      0.05      1.6255       51.236       26.979   
                                                      0.0536      0.0006         1.15   

  Sample 6                    495.00        520.00      0.07      1.6255       51.437       25.556   
  Sample 6                    495.00        520.00      0.08      1.6255       51.968       25.264   
                                                      0.0774      0.0055         7.06   

  Sample 7                    495.00        520.00      0.09      1.6255       51.428       24.533   
  Sample 7                    495.00        520.00      0.09      1.6255       51.052       24.163   
                                                      0.0894      0.0020         2.22   

  Sample 8                    495.00        520.00      0.09      1.6255       51.327       24.515   
  Sample 8                    495.00        520.00      0.09      1.6255       51.512       24.433   
                                                      0.0888      0.0018         1.97   

  Sample 9                    495.00        520.00      0.09      1.6255       51.441       24.523   
  Sample 9                    495.00        520.00      0.10      1.6255       51.661       23.838   
                                                      0.0941      0.0083         8.79   

  Sample 10                   495.00        520.00      0.10      1.6255       51.340       23.509   
  Sample 10                   495.00        520.00      0.10      1.6255       50.869       23.761   
                                                      0.0992      0.0051         5.10   

  Sample 11                   495.00        520.00      0.15      1.6255       51.656       20.630   
  Sample 11                   495.00        520.00      0.15      1.6255       51.326       20.663   
                                                      0.1512      0.0021         1.38   

  Sample 12                   495.00        520.00      0.15      1.6255       51.436       20.888   
  Sample 12                   495.00        520.00      0.14      1.6255       51.133       20.880   
                                                      0.1455      0.0014         0.99   

  Sample 13                   495.00        520.00      0.17      1.6255       49.012       18.680   
  Sample 13                   495.00        520.00      0.17      1.6255       49.100       18.763   
                                                      0.1694      0.0007         0.41   

  Sample 14                   495.00        520.00      0.18      1.6255       47.641       17.683   
  Sample 14                   495.00        520.00      0.18      1.6255       47.495       17.740   
                                                      0.1786      0.0017         0.93   

  Sample 15                   495.00        520.00      0.19      1.6255       47.403       17.006   
  Sample 15                   495.00        520.00      0.19      1.6255       47.297       17.149   
                                                      0.1904      0.0028         1.47   

Read sequence cancelled

Results Flags Legend
R = Repeat reading               @ = Over-range                                       
