# Supplementary material for: The Shu complex prevents mutagenesis and cytotoxicity of single-strand specific alkylation lesions
Source: eLife. 2021 Nov 1;10:e68080. doi: 10.7554/eLife.68080 (PMC8610418; doi:10.7554/eLife.68080)
Supplement: Figure 4—source data 3. [file elife-68080-fig4-data3.zip › 9_2_20215nM3MeCCsm2Psy3T2.RTF]

Advanced Reads Report

Report Time : Thu 02 Sep 04:07:03 PM 2021
Batch: C:\Documents and Settings\BEN\Desktop\Sarah\9_2_20215nM3MeCCsm2Psy3T2.FBAB
Software Version: 1.1(132)
Operator: 


Instrument Parameters

Instrument                        Cary Eclipse                                                        
Instrument Serial Number          FL0908M003                                                          
Data mode                         Fluorescence                                                        
User Result                       execute("AutoPolarizationCollect.ADL")                              
Ex. Slit (nm)                     10                                                                  
Em. Slit (nm)                     10                                                                  
Ave Time (sec)                    2.0000                                                              
Excitation filter                 Auto                                                                
Emission filter                   Auto                                                                
PMT Voltage (V)                   700                                                                 
Multicell holder                  Multicell                                                           
 Multi zero                       ON                                                                  
Device                                                                                                
 Set temperature (°C)             25.00                                                               
 Monitor                          Block                                                               
Replicates                        OFF                                                                 
Sample averaging                  Duplicate                                                           
Comments:

 
G-Factor
 
 Instrument                5
 Data mode                 Fluorescence
 Ex. Slit (nm)             10
 Em. slit (nm)             10
 Ave. time(s)              2.00000

Ex. WL (nm)   Em. WL (nm)   G-Factor    Int(HV) (a.u)   Int(HH) (a.u.)   
_________________________________________________________________________
     495.00        520.00      1.6077         440.517          274.012   
 
Analysis
Collection time                  9/2/2021 4:07:18 PM                                  
 
Anisotropy
 
     Sample Name         Ex. WL (nm)   Em. WL (nm)      r      G-Factor      Int(VV)      Int(VH)    
_____________________________________________________________________________________________________
  Sample 1                    495.00        520.00      0.04      1.6077       42.180       23.184   
  Sample 1                    495.00        520.00      0.04      1.6077       42.153       23.125   
                                                      0.0424      0.0005         1.09   

  Sample 2                    495.00        520.00      0.04      1.6077       42.183       23.194   
  Sample 2                    495.00        520.00      0.05      1.6077       42.617       22.929   
                                                      0.0457      0.0053        11.66   

  Sample 3                    495.00        520.00      0.05      1.6077       42.093       22.804   
  Sample 3                    495.00        520.00      0.05      1.6077       41.788       22.493   
                                                      0.0482      0.0016         3.30   

  Sample 4                    495.00        520.00      0.05      1.6077       41.979       22.478   
  Sample 4                    495.00        520.00      0.05      1.6077       42.313       22.496   
                                                      0.0524      0.0018         3.36   

  Sample 5                    495.00        520.00      0.06      1.6077       42.067       22.162   
  Sample 5                    495.00        520.00      0.06      1.6077       41.800       21.974   
                                                      0.0572      0.0005         0.93   

  Sample 6                    495.00        520.00      0.07      1.6077       42.381       21.458   
  Sample 6                    495.00        520.00      0.07      1.6077       42.683       21.521   
                                                      0.0715      0.0010         1.44   

  Sample 7                    495.00        520.00      0.09      1.6077       42.761       20.485   
  Sample 7                    495.00        520.00      0.09      1.6077       42.505       20.628   
                                                      0.0881      0.0033         3.73   

  Sample 8                    495.00        520.00      0.10      1.6077       42.595       20.115   
  Sample 8                    495.00        520.00      0.10      1.6077       42.676       19.951   
                                                      0.0974      0.0026         2.62   

  Sample 9                    495.00        520.00      0.10      1.6077       43.113       20.221   
  Sample 9                    495.00        520.00      0.10      1.6077       42.572       19.944   
                                                      0.0983      0.0003         0.31   

  Sample 10                   495.00        520.00      0.14      1.6077       43.377       18.062   
  Sample 10                   495.00        520.00      0.15      1.6077       43.639       17.922   
                                                      0.1439      0.0036         2.49   

  Sample 11                   495.00        520.00      0.17      1.6077       44.155       16.830   
  Sample 11                   495.00        520.00      0.17      1.6077       43.850       16.723   
                                                      0.1739      0.0001         0.08   

  Sample 12                   495.00        520.00      0.18      1.6077       43.536       16.428   
  Sample 12                   495.00        520.00      0.18      1.6077       43.719       16.312   
                                                      0.1798      0.0030         1.65   

  Sample 13                   495.00        520.00      0.19      1.6077       42.605       15.723   
  Sample 13                   495.00        520.00      0.19      1.6077       42.752       15.722   
                                                      0.1866      0.0009         0.50   

  Sample 14                   495.00        520.00      0.19      1.6077       43.101       15.575   
  Sample 14                   495.00        520.00      0.19      1.6077       42.589       15.432   
                                                      0.1933      0.0007         0.37   

  Sample 15                   495.00        520.00      0.20      1.6077       40.766       14.646   
  Sample 15                   495.00        520.00      0.20      1.6077       41.002       14.568   
                                                      0.1981      0.0029         1.48   

Read sequence cancelled

Results Flags Legend
R = Repeat reading               @ = Over-range                                       
