# Supplementary material for: The Shu complex prevents mutagenesis and cytotoxicity of single-strand specific alkylation lesions
Source: eLife. 2021 Nov 1;10:e68080. doi: 10.7554/eLife.68080 (PMC8610418; doi:10.7554/eLife.68080)
Supplement: Figure 4—source data 3. [file elife-68080-fig4-data3.zip › 9_2_20215nM3MeCCsm2Psy3T3.RTF]

Advanced Reads Report

Report Time : Thu 02 Sep 08:21:46 PM 2021
Batch: C:\Documents and Settings\BEN\Desktop\Sarah\9_2_20215nM3MeCCsm2Psy3T3.FBAB
Software Version: 1.1(132)
Operator: 


Instrument Parameters

Instrument                        Cary Eclipse                                                        
Instrument Serial Number          FL0908M003                                                          
Data mode                         Fluorescence                                                        
User Result                       execute("AutoPolarizationCollect.ADL")                              
Ex. Slit (nm)                     10                                                                  
Em. Slit (nm)                     10                                                                  
Ave Time (sec)                    2.0000                                                              
Excitation filter                 Auto                                                                
Emission filter                   Auto                                                                
PMT Voltage (V)                   700                                                                 
Multicell holder                  Multicell                                                           
 Multi zero                       ON                                                                  
Device                                                                                                
 Set temperature (°C)             25.00                                                               
 Monitor                          Block                                                               
Replicates                        OFF                                                                 
Sample averaging                  Duplicate                                                           
Comments:

 
G-Factor
 
 Instrument                5
 Data mode                 Fluorescence
 Ex. Slit (nm)             10
 Em. slit (nm)             10
 Ave. time(s)              2.00000

Ex. WL (nm)   Em. WL (nm)   G-Factor    Int(HV) (a.u)   Int(HH) (a.u.)   
_________________________________________________________________________
     495.00        520.00      1.6312         382.453          234.456   
 
Analysis
Collection time                  9/2/2021 8:23:26 PM                                  
 
Anisotropy
 
     Sample Name         Ex. WL (nm)   Em. WL (nm)      r      G-Factor      Int(VV)      Int(VH)    
_____________________________________________________________________________________________________
  Sample 1                    495.00        520.00      0.03      1.6312       36.423       20.239   
  Sample 1                    495.00        520.00      0.04      1.6312       36.585       20.079   
                                                      0.0354      0.0030         8.52   

  Sample 2                    495.00        520.00      0.05      1.6312       35.668       19.074   
  Sample 2                    495.00        520.00      0.04      1.6312       35.222       19.229   
                                                      0.0429      0.0051        11.79   

  Sample 3                    495.00        520.00      0.05      1.6312       35.304       18.679   
  Sample 3                    495.00        520.00      0.05      1.6312       34.929       18.619   
                                                      0.0489      0.0018         3.75   

  Sample 4                    495.00        520.00      0.05      1.6312       34.619       18.165   
  Sample 4                    495.00        520.00      0.05      1.6312       34.511       18.235   
                                                      0.0519      0.0017         3.32   

  Sample 5                    495.00        520.00      0.06      1.6312       35.033       18.256   
  Sample 5                    495.00        520.00      0.05      1.6312       34.799       18.198   
                                                      0.0549      0.0009         1.60   

  Sample 6                    495.00        520.00      0.08      1.6312       35.251       17.380   
  Sample 6                    495.00        520.00      0.07      1.6312       35.128       17.473   
                                                      0.0735      0.0022         3.02   

  Sample 7                    495.00        520.00      0.09      1.6312       35.029       16.656   
  Sample 7                    495.00        520.00      0.09      1.6312       35.255       16.857   
                                                      0.0870      0.0014         1.61   

  Sample 8                    495.00        520.00      0.10      1.6312       35.952       16.682   
  Sample 8                    495.00        520.00      0.10      1.6312       35.607       16.418   
                                                      0.0978      0.0016         1.64   

  Sample 9                    495.00        520.00      0.09      1.6312       35.536       16.590   
  Sample 9                    495.00        520.00      0.11      1.6312       36.075       16.333   
                                                      0.1000      0.0078         7.81   

  Sample 10                   495.00        520.00      0.15      1.6312       36.054       14.466   
  Sample 10                   495.00        520.00      0.15      1.6312       35.810       14.384   
                                                      0.1494      0.0003         0.19   

  Sample 11                   495.00        520.00      0.17      1.6312       35.503       13.433   
  Sample 11                   495.00        520.00      0.17      1.6312       35.435       13.405   
                                                      0.1713      0.0000         0.03   

  Sample 12                   495.00        520.00      0.19      1.6312       34.921       12.673   
  Sample 12                   495.00        520.00      0.19      1.6312       34.471       12.496   
                                                      0.1870      0.0003         0.15   

  Sample 13                   495.00        520.00      0.20      1.6312       34.067       12.053   
  Sample 13                   495.00        520.00      0.21      1.6312       34.432       11.831   
                                                      0.2018      0.0077         3.83   

  Sample 14                   495.00        520.00      0.21      1.6312       33.473       11.379   
  Sample 14                   495.00        520.00      0.20      1.6312       33.018       11.490   
                                                      0.2069      0.0062         2.99   

Read sequence cancelled

Results Flags Legend
R = Repeat reading               @ = Over-range                                       
