# Supplementary material for: The Shu complex prevents mutagenesis and cytotoxicity of single-strand specific alkylation lesions
Source: eLife. 2021 Nov 1;10:e68080. doi: 10.7554/eLife.68080 (PMC8610418; doi:10.7554/eLife.68080)
Supplement: Figure 4—source data 3. [file elife-68080-fig4-data3.zip › 9_2_20215nMWTTCsm2Psy3T2.RTF]

Advanced Reads Report

Report Time : Thu 02 Sep 11:57:41 AM 2021
Batch: C:\Documents and Settings\BEN\Desktop\Sarah\9_2_20215nMWTTCsm2Psy3T2.FBAB
Software Version: 1.1(132)
Operator: 


Instrument Parameters

Instrument                        Cary Eclipse                                                        
Instrument Serial Number          FL0908M003                                                          
Data mode                         Fluorescence                                                        
User Result                       execute("AutoPolarizationCollect.ADL")                              
Ex. Slit (nm)                     10                                                                  
Em. Slit (nm)                     10                                                                  
Ave Time (sec)                    2.0000                                                              
Excitation filter                 Auto                                                                
Emission filter                   Auto                                                                
PMT Voltage (V)                   800                                                                 
Multicell holder                  Multicell                                                           
 Multi zero                       ON                                                                  
Device                                                                                                
 Set temperature (°C)             25.00                                                               
 Monitor                          Block                                                               
Replicates                        OFF                                                                 
Sample averaging                  Duplicate                                                           
Comments:

 
G-Factor
 
 Instrument                5
 Data mode                 Fluorescence
 Ex. Slit (nm)             10
 Em. slit (nm)             10
 Ave. time(s)              2.00000

Ex. WL (nm)   Em. WL (nm)   G-Factor    Int(HV) (a.u)   Int(HH) (a.u.)   
_________________________________________________________________________
     495.00        520.00      1.3272          44.057           33.196   
 
Analysis
Collection time                  9/2/2021 11:58:07 AM                                 
 
Anisotropy
 
     Sample Name         Ex. WL (nm)   Em. WL (nm)      r      G-Factor      Int(VV)      Int(VH)    
_____________________________________________________________________________________________________
  Sample 1                    495.00        520.00      0.27      1.3272        6.530        2.316   
  Sample 1                    495.00        520.00      0.30      1.3272        6.659        2.174   
                                                      0.2882      0.0219         7.60   

  Sample 2                    495.00        520.00      0.29      1.3272        6.604        2.209   
  Sample 2                    495.00        520.00      0.30      1.3272        6.865        2.239   
                                                      0.2993      0.0067         2.23   

  Sample 3                    495.00        520.00      0.31      1.3272        6.837        2.205   
  Sample 3                    495.00        520.00      0.29      1.3272        6.852        2.289   
                                                      0.3015      0.0093         3.08   

  Sample 4                    495.00        520.00      0.32      1.3272        6.773        2.121   
  Sample 4                    495.00        520.00      0.31      1.3272        6.892        2.207   
                                                      0.3150      0.0059         1.87   

  Sample 5                    495.00        520.00      0.33      1.3272        6.815        2.080   
  Sample 5                    495.00        520.00      0.29      1.3272        6.650        2.231   
                                                      0.3111      0.0249         8.02   

  Sample 6                    495.00        520.00      0.32      1.3272        6.844        2.114   
  Sample 6                    495.00        520.00      0.34      1.3272        7.023        2.103   
                                                      0.3300      0.0082         2.48   

  Sample 7                    495.00        520.00      0.36      1.3272        7.003        1.949   
  Sample 7                    495.00        520.00      0.31      1.3272        6.838        2.224   
                                                      0.3339      0.0409        12.24   

  Sample 8                    495.00        520.00      0.34      1.3272        7.027        2.055   
  Sample 8                    495.00        520.00      0.36      1.3272        7.122        1.985   
                                                      0.3534      0.0125         3.55   

  Sample 9                    495.00        520.00      0.36      1.3272        7.020        1.956   
  Sample 9                    495.00        520.00      0.37      1.3272        6.976        1.902   
                                                      0.3662      0.0056         1.54   

  Sample 10                   495.00        520.00      0.39      1.3272        7.433        1.919   
  Sample 10                   495.00        520.00      0.40      1.3272        7.572        1.882   
                                                      0.3969      0.0098         2.46   

  Sample 11                   495.00        520.00      0.42      1.3272        7.822        1.842   
  Sample 11                   495.00        520.00      0.42      1.3272        7.743        1.853   
                                                      0.4202      0.0042         0.99   

  Sample 12                   495.00        520.00      0.44      1.3272        8.097        1.834   
  Sample 12                   495.00        520.00      0.41      1.3272        7.512        1.826   
                                                      0.4242      0.0178         4.20   

  Sample 13                   495.00        520.00      0.40      1.3272        7.598        1.912   
  Sample 13                   495.00        520.00      0.43      1.3272        7.793        1.794   
                                                      0.4153      0.0225         5.41   

  Sample 14                   495.00        520.00      0.43      1.3272        7.593        1.778   
  Sample 14                   495.00        520.00      0.44      1.3272        7.756        1.733   
                                                      0.4333      0.0116         2.67   

Read sequence cancelled

Results Flags Legend
R = Repeat reading               @ = Over-range                                       
