# Supplementary material for: The Shu complex prevents mutagenesis and cytotoxicity of single-strand specific alkylation lesions
Source: eLife. 2021 Nov 1;10:e68080. doi: 10.7554/eLife.68080 (PMC8610418; doi:10.7554/eLife.68080)
Supplement: Figure 4—source data 3. [file elife-68080-fig4-data3.zip › 9_2_20215nM1MeACsm2Psy3T2.RTF]

Advanced Reads Report

Report Time : Thu 02 Sep 07:02:37 PM 2021
Batch: C:\Documents and Settings\BEN\Desktop\Sarah\9_2_20215nM1MeACsm2Psy3T2.FBAB
Software Version: 1.1(132)
Operator: 


Instrument Parameters

Instrument                        Cary Eclipse                                                        
Instrument Serial Number          FL0908M003                                                          
Data mode                         Fluorescence                                                        
User Result                       execute("AutoPolarizationCollect.ADL")                              
Ex. Slit (nm)                     10                                                                  
Em. Slit (nm)                     10                                                                  
Ave Time (sec)                    2.0000                                                              
Excitation filter                 Auto                                                                
Emission filter                   Auto                                                                
PMT Voltage (V)                   700                                                                 
Multicell holder                  Multicell                                                           
 Multi zero                       ON                                                                  
Device                                                                                                
 Set temperature (°C)             25.00                                                               
 Monitor                          Block                                                               
Replicates                        OFF                                                                 
Sample averaging                  Duplicate                                                           
Comments:

 
G-Factor
 
 Instrument                5
 Data mode                 Fluorescence
 Ex. Slit (nm)             10
 Em. slit (nm)             10
 Ave. time(s)              2.00000

Ex. WL (nm)   Em. WL (nm)   G-Factor    Int(HV) (a.u)   Int(HH) (a.u.)   
_________________________________________________________________________
     495.00        520.00      1.6403         659.879          402.294   
 
Analysis
Collection time                  9/2/2021 7:03:15 PM                                  
 
Anisotropy
 
     Sample Name         Ex. WL (nm)   Em. WL (nm)      r      G-Factor      Int(VV)      Int(VH)    
_____________________________________________________________________________________________________
  Sample 1                    495.00        520.00      0.04      1.6403       61.565       33.788   
  Sample 1                    495.00        520.00      0.04      1.6403       61.864       33.750   
                                                      0.0367      0.0015         3.97   

  Sample 2                    495.00        520.00      0.04      1.6403       56.653       31.030   
  Sample 2                    495.00        520.00      0.04      1.6403       56.956       31.274   
                                                      0.0359      0.0006         1.70   

  Sample 3                    495.00        520.00      0.04      1.6403       57.044       30.910   
  Sample 3                    495.00        520.00      0.04      1.6403       56.937       30.819   
                                                      0.0402      0.0003         0.65   

  Sample 4                    495.00        520.00      0.04      1.6403       56.670       30.537   
  Sample 4                    495.00        520.00      0.04      1.6403       56.363       30.603   
                                                      0.0406      0.0019         4.57   

  Sample 5                    495.00        520.00      0.04      1.6403       57.083       30.686   
  Sample 5                    495.00        520.00      0.04      1.6403       57.395       30.815   
                                                      0.0430      0.0003         0.72   

  Sample 6                    495.00        520.00      0.06      1.6403       58.350       29.614   
  Sample 6                    495.00        520.00      0.06      1.6403       57.652       29.656   
                                                      0.0605      0.0033         5.52   

  Sample 7                    495.00        520.00      0.07      1.6403       58.285       29.153   
  Sample 7                    495.00        520.00      0.07      1.6403       58.360       29.282   
                                                      0.0674      0.0008         1.16   

  Sample 8                    495.00        520.00      0.08      1.6403       58.582       28.015   
  Sample 8                    495.00        520.00      0.08      1.6403       58.139       28.008   
                                                      0.0826      0.0018         2.24   

  Sample 9                    495.00        520.00      0.08      1.6403       58.045       28.111   
  Sample 9                    495.00        520.00      0.08      1.6403       58.345       28.035   
                                                      0.0808      0.0020         2.45   

  Sample 10                   495.00        520.00      0.14      1.6403       58.700       24.008   
  Sample 10                   495.00        520.00      0.14      1.6403       58.841       24.021   
                                                      0.1409      0.0005         0.35   

  Sample 11                   495.00        520.00      0.17      1.6403       58.281       22.200   
  Sample 11                   495.00        520.00      0.16      1.6403       58.272       22.467   
                                                      0.1645      0.0032         1.92   

  Sample 12                   495.00        520.00      0.18      1.6403       56.784       20.892   
  Sample 12                   495.00        520.00      0.18      1.6403       56.789       20.895   
                                                      0.1796      0.0000         0.01   

  Sample 13                   495.00        520.00      0.19      1.6403       55.111       19.711   
  Sample 13                   495.00        520.00      0.19      1.6403       54.914       19.730   
                                                      0.1893      0.0012         0.63   

  Sample 14                   495.00        520.00      0.19      1.6403       52.707       18.863   
  Sample 14                   495.00        520.00      0.19      1.6403       53.461       18.885   
                                                      0.1924      0.0034         1.79   

  Sample 15                   495.00        520.00      0.20      1.6403       52.206       18.122   
  Sample 15                   495.00        520.00      0.20      1.6403       52.107       18.224   
                                                      0.1999      0.0020         1.00   

  Sample 16                   495.00        520.00      0.20      1.6403       52.207       18.239   
Read sequence cancelled

Results Flags Legend
R = Repeat reading               @ = Over-range                                       
