# Supplementary material for: The Shu complex prevents mutagenesis and cytotoxicity of single-strand specific alkylation lesions
Source: eLife. 2021 Nov 1;10:e68080. doi: 10.7554/eLife.68080 (PMC8610418; doi:10.7554/eLife.68080)
Supplement: Figure 4—source data 3. [file elife-68080-fig4-data3.zip › 9_2_20215nM1MeACsm2Psy3T1.RTF]

Advanced Reads Report

Report Time : Thu 02 Sep 05:05:29 PM 2021
Batch: C:\Documents and Settings\BEN\Desktop\Sarah\9_2_20215nM1MeACsm2Psy3T1.FBAB
Software Version: 1.1(132)
Operator: 


Instrument Parameters

Instrument                        Cary Eclipse                                                        
Instrument Serial Number          FL0908M003                                                          
Data mode                         Fluorescence                                                        
User Result                       execute("AutoPolarizationCollect.ADL")                              
Ex. Slit (nm)                     10                                                                  
Em. Slit (nm)                     10                                                                  
Ave Time (sec)                    2.0000                                                              
Excitation filter                 Auto                                                                
Emission filter                   Auto                                                                
PMT Voltage (V)                   700                                                                 
Multicell holder                  Multicell                                                           
 Multi zero                       ON                                                                  
Device                                                                                                
 Set temperature (°C)             25.00                                                               
 Monitor                          Block                                                               
Replicates                        OFF                                                                 
Sample averaging                  Duplicate                                                           
Comments:

 
G-Factor
 
 Instrument                5
 Data mode                 Fluorescence
 Ex. Slit (nm)             10
 Em. slit (nm)             10
 Ave. time(s)              2.00000

Ex. WL (nm)   Em. WL (nm)   G-Factor    Int(HV) (a.u)   Int(HH) (a.u.)   
_________________________________________________________________________
     495.00        520.00      1.6281         605.002          371.600   
 
Analysis
Collection time                  9/2/2021 5:06:36 PM                                  
 
Anisotropy
 
     Sample Name         Ex. WL (nm)   Em. WL (nm)      r      G-Factor      Int(VV)      Int(VH)    
_____________________________________________________________________________________________________
  Sample 1                    495.00        520.00      0.04      1.6281       58.203       31.532   
  Sample 1                    495.00        520.00      0.04      1.6281       57.410       31.686   
                                                      0.0395      0.0045        11.50   

  Sample 2                    495.00        520.00      0.04      1.6281       57.259       31.547   
  Sample 2                    495.00        520.00      0.05      1.6281       57.385       30.839   
                                                      0.0412      0.0061        14.80   

  Sample 3                    495.00        520.00      0.05      1.6281       58.911       31.222   
  Sample 3                    495.00        520.00      0.05      1.6281       58.569       30.687   
                                                      0.0523      0.0028         5.41   

  Sample 4                    495.00        520.00      0.05      1.6281       60.098       31.619   
  Sample 4                    495.00        520.00      0.05      1.6281       59.934       31.791   
                                                      0.0514      0.0020         3.92   

  Sample 5                    495.00        520.00      0.06      1.6281       60.697       31.419   
  Sample 5                    495.00        520.00      0.06      1.6281       60.591       31.629   
                                                      0.0571      0.0021         3.65   

  Sample 6                    495.00        520.00      0.06      1.6281       59.359       30.550   
  Sample 6                    495.00        520.00      0.06      1.6281       59.501       30.657   
                                                      0.0604      0.0003         0.45   

  Sample 7                    495.00        520.00      0.07      1.6281       59.608       29.838   
  Sample 7                    495.00        520.00      0.08      1.6281       60.008       29.485   
                                                      0.0736      0.0046         6.31   

  Sample 8                    495.00        520.00      0.08      1.6281       62.400       30.466   
  Sample 8                    495.00        520.00      0.08      1.6281       62.163       30.541   
                                                      0.0781      0.0016         2.01   

  Sample 9                    495.00        520.00      0.09      1.6281       63.392       29.847   
  Sample 9                    495.00        520.00      0.08      1.6281       62.499       30.046   
                                                      0.0884      0.0053         5.95   

  Sample 10                   495.00        520.00      0.14      1.6281       66.592       27.230   
  Sample 10                   495.00        520.00      0.15      1.6281       66.793       26.984   
                                                      0.1456      0.0031         2.16   

  Sample 11                   495.00        520.00      0.17      1.6281       65.187       24.695   
  Sample 11                   495.00        520.00      0.17      1.6281       65.230       24.855   
                                                      0.1705      0.0015         0.89   

  Sample 12                   495.00        520.00      0.19      1.6281       63.642       23.052   
  Sample 12                   495.00        520.00      0.19      1.6281       63.532       23.162   
                                                      0.1870      0.0017         0.92   

  Sample 13                   495.00        520.00      0.19      1.6281       61.573       22.292   
  Sample 13                   495.00        520.00      0.19      1.6281       61.388       21.939   
                                                      0.1908      0.0034         1.79   

  Sample 14                   495.00        520.00      0.21      1.6281       49.894       17.227   
  Sample 14                   495.00        520.00      0.21      1.6281       50.208       16.934   
                                                      0.2105      0.0062         2.95   

  Sample 15                   495.00        520.00      0.19      1.6281       59.318       21.227   
  Sample 15                   495.00        520.00      0.20      1.6281       59.574       21.118   
                                                      0.1945      0.0025         1.28   

Read sequence cancelled

Results Flags Legend
R = Repeat reading               @ = Over-range                                       
