# Supplementary material for: The Shu complex prevents mutagenesis and cytotoxicity of single-strand specific alkylation lesions
Source: eLife. 2021 Nov 1;10:e68080. doi: 10.7554/eLife.68080 (PMC8610418; doi:10.7554/eLife.68080)
Supplement: Figure 4—source data 3. [file elife-68080-fig4-data3.zip › 8_19_2021for5nM3M3CplusCsm2Psy3T1.RTF]

Advanced Reads Report

Report Time : Thu 19 Aug 06:46:18 PM 2021
Batch: C:\Documents and Settings\BEN\Desktop\Sarah\8_19_2021for5nM3M3CplusCsm2Psy3T1.FBAB
Software Version: 1.1(132)
Operator: 


Instrument Parameters

Instrument                        Cary Eclipse                                                        
Instrument Serial Number          FL0908M003                                                          
Data mode                         Fluorescence                                                        
User Result                       execute("AutoPolarizationCollect.ADL")                              
Ex. Slit (nm)                     10                                                                  
Em. Slit (nm)                     10                                                                  
Ave Time (sec)                    2.0000                                                              
Excitation filter                 Auto                                                                
Emission filter                   Auto                                                                
PMT Voltage (V)                   700                                                                 
Multicell holder                  Multicell                                                           
 Multi zero                       ON                                                                  
Device                                                                                                
 Set temperature (°C)             25.00                                                               
 Monitor                          Block                                                               
Replicates                        OFF                                                                 
Sample averaging                  Duplicate                                                           
Comments:

 
G-Factor
 
 Instrument                5
 Data mode                 Fluorescence
 Ex. Slit (nm)             10
 Em. slit (nm)             10
 Ave. time(s)              2.00000

Ex. WL (nm)   Em. WL (nm)   G-Factor    Int(HV) (a.u)   Int(HH) (a.u.)   
_________________________________________________________________________
     495.00        520.00      1.4458        1000.000          691.640   
 
Analysis
Collection time                  8/19/2021 6:46:39 PM                                 
 
Anisotropy
 
     Sample Name         Ex. WL (nm)   Em. WL (nm)      r      G-Factor      Int(VV)      Int(VH)    
_____________________________________________________________________________________________________
  Sample 1                    495.00        520.00      0.07      1.4458      103.241       57.718   
  Sample 1                    495.00        520.00      0.07      1.4458      103.056       57.802   
                                                      0.0727      0.0008         1.12   

  Sample 2                    495.00        520.00      0.08      1.4458      101.126       55.844   
  Sample 2                    495.00        520.00      0.08      1.4458      101.775       56.017   
                                                      0.0782      0.0008         1.06   

  Sample 3                    495.00        520.00      0.08      1.4458       99.438       54.477   
  Sample 3                    495.00        520.00      0.08      1.4458      100.259       54.702   
                                                      0.0812      0.0010         1.27   

  Sample 4                    495.00        520.00      0.08      1.4458       97.653       53.237   
  Sample 4                    495.00        520.00      0.09      1.4458       98.306       53.118   
                                                      0.0838      0.0022         2.68   

  Sample 5                    495.00        520.00      0.08      1.4458       96.498       52.305   
  Sample 5                    495.00        520.00      0.08      1.4458       96.423       52.538   
                                                      0.0833      0.0013         1.58   

  Sample 6                    495.00        520.00      0.10      1.4458       98.132       51.288   
  Sample 6                    495.00        520.00      0.09      1.4458       97.799       51.473   
                                                      0.0961      0.0018         1.85   

  Sample 7                    495.00        520.00      0.11      1.4458       99.207       50.525   
  Sample 7                    495.00        520.00      0.11      1.4458       98.805       50.345   
                                                      0.1065      0.0001         0.12   

  Sample 8                    495.00        520.00      0.11      1.4458       98.425       49.303   
  Sample 8                    495.00        520.00      0.12      1.4458       98.911       49.046   
                                                      0.1145      0.0026         2.27   

  Sample 9                    495.00        520.00      0.12      1.4458       98.101       48.276   
  Sample 9                    495.00        520.00      0.12      1.4458       98.002       47.901   
                                                      0.1203      0.0017         1.45   

  Sample 10                   495.00        520.00      0.17      1.4458      100.033       42.958   
  Sample 10                   495.00        520.00      0.17      1.4458      100.196       42.963   
                                                      0.1694      0.0004         0.23   

  Sample 11                   495.00        520.00      0.19      1.4458       99.232       40.356   
  Sample 11                   495.00        520.00      0.19      1.4458       98.910       40.361   
                                                      0.1887      0.0009         0.47   

  Sample 12                   495.00        520.00      0.20      1.4458       96.098       37.665   
  Sample 12                   495.00        520.00      0.20      1.4458       95.541       37.746   
                                                      0.2016      0.0021         1.04   

  Sample 13                   495.00        520.00      0.21      1.4458       92.957       35.741   
  Sample 13                   495.00        520.00      0.21      1.4458       92.933       35.667   
                                                      0.2106      0.0005         0.23   

Read sequence cancelled

Results Flags Legend
R = Repeat reading               @ = Over-range                                       
